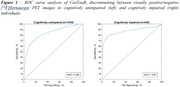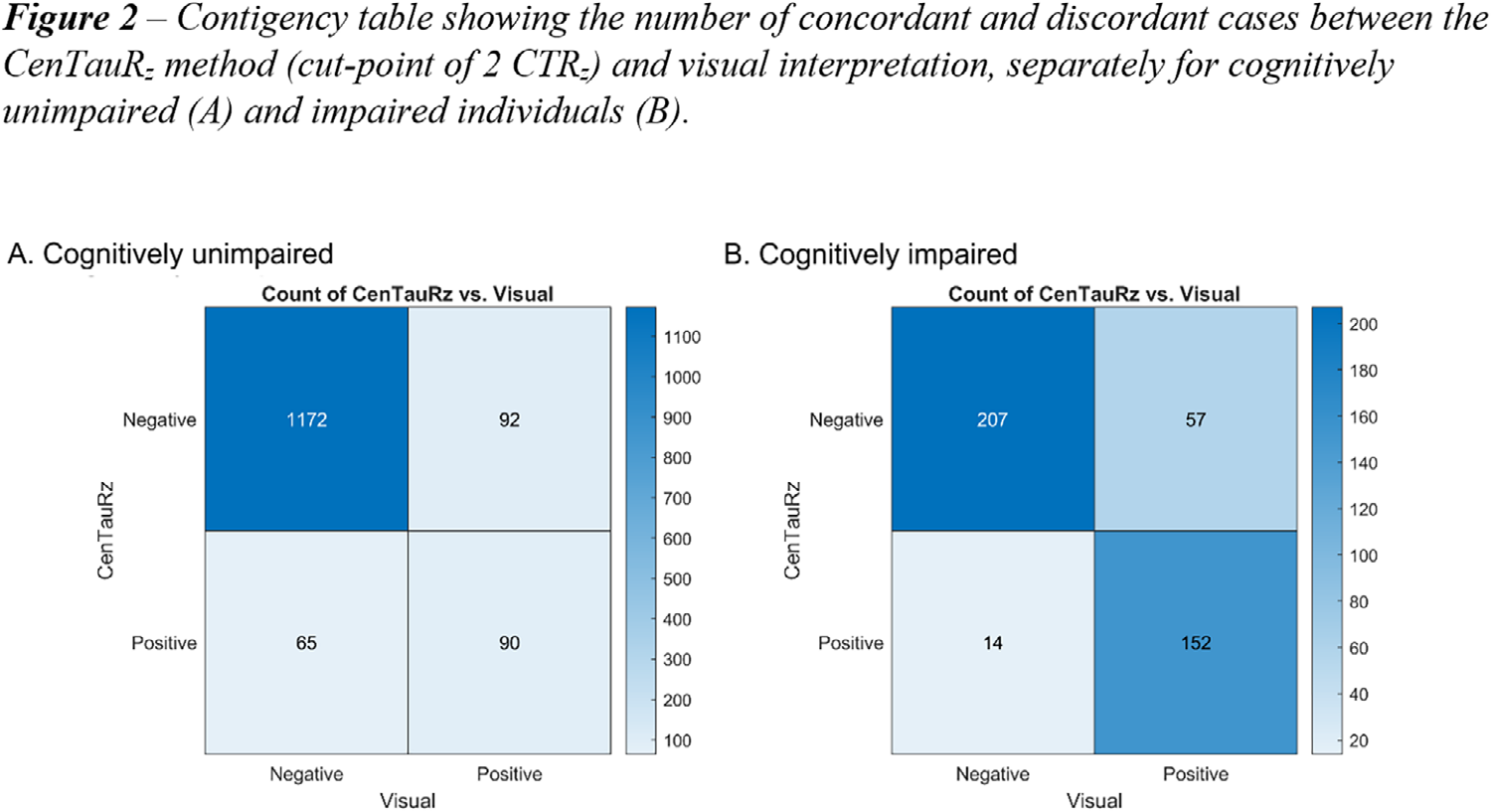# Concordance between visual interpretation of [^18^F]flortaucipir pet images and the Universal centaur scale

**DOI:** 10.1002/alz.095751

**Published:** 2025-01-09

**Authors:** Alexis Moscoso, Martijn van Essen, Isminni Mainta, Valle Camacho, Omar Rodríguez‐Fonseca, Jesús Silva‐Rodríguez, Andrés Perissinotti, Nicolai Franzmeier, Michel J. Grothe, Giovanni B Frisoni, Valentina Garibotto, Michael Schöll

**Affiliations:** ^1^ Department of Psychiatry and Neurochemistry, Institute of Neuroscience and Physiology, University of Gothenburg, Mölndal Sweden; ^2^ Wallenberg Centre for Molecular and Translational Medicine, University of Gothenburg, Gothenburg Sweden; ^3^ Department of Clinical Physiology, Sahlgrenska University Hospital, Gothenburg Sweden; ^4^ Division of Nuclear Medicine, Geneva University Hospitals, Geneva Switzerland; ^5^ Nuclear Medicine Department, Hospital de la Santa Creu i Sant Pau, Barcelona Spain; ^6^ Nuclear Medicine Department, Lucus Augusti University Hospital (HULA), 27003 Lugo, Spain., Lugo, Galicia Spain; ^7^ Reina Sofia Alzheimer Center, CIEN Foundation, ISCIII, Madrid, Madrid Spain; ^8^ Centro de Investigación Biomédica en Red de Bioingeniería, Biomateriales y Nanomedicina (CIBER‐BBN), Madrid Spain; ^9^ Nuclear Medicine Department, Hospital Clínic Barcelona, Barcelona Spain; ^10^ University of Gothenburg, The Sahlgrenska Academy, Institute of Neuroscience and Physiology, Psychiatry and Neurochemistry, Gothenburg Sweden; ^11^ Institute for Stroke and Dementia Research (ISD), University Hospital, LMU, Munich, Bayern Germany; ^12^ Munich Cluster for Systems Neurology (SyNergy), Munich, Bavaria Germany; ^13^ Institute for Stroke and Dementia Research, Ludwig‐Maximilians‐Universität München, LMU München, Munich Germany; ^14^ Laboratory of Neuroimaging of Aging (LANVIE), University of Geneva, Geneva Switzerland; ^15^ Geneva Memory Center, Department of Rehabilitation and Geriatrics, Geneva University Hospitals, Geneva Switzerland; ^16^ Geneva University Hospitals, Geneva Switzerland; ^17^ Faculty of Medicine, University of Geneva, Geneva Switzerland; ^18^ Department of Psychiatry and Neurochemistry, Institute of Neuroscience and Physiology, The Sahlgrenska Academy, University of Gothenburg, Mölndal Sweden

## Abstract

**Background:**

Positron emission tomography (PET) imaging with [^18^F]flortaucipir allows for in‐vivo visualization of aggregated tau in Alzheimer’s disease (AD). The FDA‐approved label for [^18^F]flortaucipir PET provides a standardized, clinically applicable definition of tau‐PET positivity by visual interpretation. Here, we studied the concordance between this clinically approved definition of tau PET positivity and a recently proposed universal scale—CenTauR—for the standardized quantification of abnormal tau aggregates.

**Method:**

We included 1849 participants (cognitively unimpaired [CU] and impaired [CI, MCI or AD dementia]) from four cohorts (A4 study, ADNI, OASIS‐3, and HABS) with available [^18^F]flortaucipir PET (mean age: 72.2 y, 55% females). Three trained readers scored each [^18^F]flortaucipir PET scan as positive/negative using an FDA‐approved visual interpretation method. Visual reads were compared to [^18^F]flortaucipir PET quantification using the Universal CenTauR_z_ scale. Specifically, we analysed concordance by 1) performing a receiver operating characteristic (ROC) curve analysis of continuous CenTauR_z_ (CTR_z_) values discriminating between positive/negative visual reads; and 2) computing sensitivity and specificity when using a previously proposed cut‐point of 2 CTR_z_.

**Result:**

Concordance between visual interpretation and continuous CTR_z_ measures was limited (AUC = 0.82 for CU and AUC = 0.87 for CI, Figure 1). When using a previously proposed cut‐point of 2 CTR_z_, this quantitative approach yielded a sensitivity of 49% [95% CI, 42% to 57%] and a specificity of 95% [93% to 96%] in CU individuals and a sensitivity of 73% [95% CI, 66% to 79%] and a specificity of 94% [95% CI, 90% to 96%] in CI individuals. Visual‐positive, CenTauR_z_‐negative individuals were more frequently Aβ‐positive than visual‐negative, CenTauR_z_‐positive individuals (87% vs 48%, *p<*0.001).

**Conclusion:**

Visual interpretation of [^18^F]flortaucipir PET images and quantification using the universal CenTauR_z_ scale exhibited limited agreement, particularly among CU individuals. Tau‐PET positivity based on visual interpretation aligns better with the presence of Aβ‐pathology. Visual interpretation seems to be more sensitive to early tau aggregation compared to universal CenTauR_z_ quantification. Future analyses will compare clinical outcomes associated to each definition of tau‐PET positivity.